# Clinical Impact of Macrotroponin on Immunometric Troponin Assays: A Cross-Analytical Case Evaluation

**DOI:** 10.3390/ijms27021117

**Published:** 2026-01-22

**Authors:** Michela Salvatici, Chiara Corrado, Rita Passerini, Monica Gaimarri, Delia Francesca Sansico, Ludovica Barile, Francesco Bandera, Lorenzo Drago

**Affiliations:** 1 UOC Laboratory of Clinical Medicine with Specialized Areas, IRCCS MultiMedica Hospital, 20138 Milan, Italy; michela.salvatici@multimedica.it (M.S.); chiara.corrado@multimedica.it (C.C.); monica.gaimarri@multimedica.it (M.G.); deliafrancesca.sansico@multimedica.it (D.F.S.); 2 Clinical Laboratory with Specialized Areas, IRCCS European Institute of Oncology, 20141 Milan, Italy; rita.passerini@ieo.it; 3 Cardiology Unit, IRCCS MultiMedica, 20099 Milan, Italy; ludovica.barile@multimedica.it (L.B.); francesco.bandera@unimi.it (F.B.); 4 Department of Biomedical Sciences for Health, University of Milan, 20133 Milan, Italy; 5 Clinical Microbiology and Microbiome Laboratory, Department of Biomedical Sciences for Health, University of Milan, 20133 Milan, Italy

**Keywords:** high-sensitivity cardiac troponin, macrotroponin, analytical interference, immunoglobulin–troponin complex, PEG precipitation, cross-platform comparison

## Abstract

High-sensitivity cardiac troponin assays are susceptible to analytical interferences, including macrotroponin, an immunoglobulin-bound complex that may cause persistent, non-dynamic, and assay-dependent elevations. This paper describes the case of a 74-year-old woman with hypertension who presented with intermittent mild chest discomfort and exertional dyspnea and exhibited persistently elevated but stable cardiac troponin I concentrations over several months, in the absence of clinical, electrocardiographic, or imaging evidence of myocardial injury. Orthogonal testing revealed discordant results across different assay platforms, and polyethylene-glycol (PEG) precipitation resulted in a substantial signal reduction, confirming macrotroponin as the source of assay-dependent analytical interference. Recognizing this phenomenon is crucial to avoid misdiagnosis, unnecessary diagnostic procedures and inappropriate management in patients with isolated troponin elevation. In conclusion, this report provides evidence-based recommendations on the optimal diagnostic strategies and laboratory approaches to adopt in cases of suspected macrotroponin-mediated interference in high-sensitivity cardiac troponin assays.

## 1. Introduction

Cardiac troponins are regulatory proteins essential to the contractile function of striated muscle. The troponin complex comprises three subunits—C, I, and T—of which cardiac-specific isoforms of troponin I (cTnI) and troponin T (cTnT) are released into the bloodstream following cardiomyocyte injury [1]. Owing to their specificity and high diagnostic accuracy, cardiac troponins represent the biomarkers of choice for detecting myocardial injury and diagnosing acute myocardial infarction, forming the biochemical foundation of contemporary diagnostic pathways [2,3,4].

The advent of high-sensitivity cardiac troponin (hs-cTn) assays has further enhanced the early identification of myocardial necrosis by enabling reliable quantification at very low concentrations, including those detectable in healthy individuals [5]. However, these immunoassays are vulnerable to several forms of analytical interference—such as heterophile antibodies, autoantibodies, macrocomplexes, and other high–molecular-weight interactions—which may result in falsely elevated, persistent, and non-dynamic troponin concentrations [1,6,7]. Among these interferences, macrotroponin, a complex formed between circulating immunoglobulins and cardiac troponin, represents one of the most challenging and under-recognized causes of assay-dependent falsely increased or decreased troponin results [8,9].

Macrotroponin is defined as a high–molecular-weight complex formed between endogenous cardiac troponin and circulating autoantibodies directed against troponin epitopes. Stable complexes formed between circulating cardiac troponin or troponin fragments may circulate either together with or independently from the cTn complexes associated with acute myocardial infarction. These immunoglobulin-bound forms—often referred to as macrotroponin I—represent high-molecular-weight complexes that can be detected depending on the specificity and architecture of the antibodies used in the immunoassay [10].

The reported prevalence of autoantibodies against cardiac troponins ranges from 0 to 12.7% for cTnI and 0–9.9% for cTnT and may be up to threefold higher among individuals with stable cardiovascular disease [11].

Although biologically inactive, macrotroponin remains immunoreactive and may be detected by any assay specific for troponin (either cTnI or cTnT), but the degree of detection varies significantly across platforms, contributing to assay-dependent discrepancies [12,13]. Depending on antibody specificity and epitope accessibility, its presence may lead to chronically increased troponin concentrations in the absence of clinical, electrocardiographic, or imaging evidence of myocardial injury or, less frequently, to reduced recovery and falsely low or negative results. Because its detectability is assay-dependent, discrepancies between platforms and the absence of a rise-and-fall kinetic pattern are key indicators of macrotroponin interference [1,9,12].

Definitive confirmation of macrotroponin interference may require a stepwise laboratory work-up. Polyethylene glycol (PEG) precipitation represents the simplest and most used screening approach in routine laboratories for the detection of high–molecular-weight complexes. However, when further characterization is required, additional techniques—such as immunoglobulin depletion (e.g., Protein A/G) or size-exclusion chromatography—can be employed to more specifically identify troponin–immunoglobulin complexes [8,14,15].

This paper describes the case of a 74-year-old woman with persistently mild and non-dynamic elevation of cTnI over several months, despite the absence of structural heart disease or inducible ischemia. Owing to the unexpected biochemical profile, an extensive analytical investigation was conducted. Troponin was measured across multiple immunoassay platforms, including high-sensitivity assays. Additionally, samples were treated with heterophile-blocking reagents and subjected to PEG precipitation to assess the presence of high–molecular-weight immune complexes. Together, these analyses showed substantial PEG-induced signal reduction and marked cross-platform discordance, confirming that the troponin elevation reflected macrotroponin rather than true myocardial injury.

This case highlights the importance of recognizing analytical interference affecting cardiac troponin measurements and underscores the critical collaboration between clinicians and laboratory specialists to avoid misdiagnosis and unnecessary interventions.

## 2. Clinical Case

A 74-year-old woman with a medical history of hypertension and rheumatoid arthritis presented to the Emergency Department of IRCCS MultiMedica, Sesto San Giovanni Hospital with intermittent, brief, stabbing chest discomfort triggered by physical effort and associated with mild exertional dyspnea. She denied symptoms at rest, palpitations, syncope, or other alarming features. Physical examination only revealed elevated blood pressure, and the electrocardiogram showed nonspecific repolarization abnormalities without evidence of acute ischemia after prolonged clinical observation.

The patient reported that multiple laboratory assessments performed over the years had consistently shown chronically elevated creatine kinase (CK) levels, with values of 425 U/L and 618 U/L, subsequently returning to near-normal concentrations (179–176 U/L; reference range 20–180 U/L). An unexpected elevation of cardiac troponin I was subsequently detected using the VIDAS^®^ assay (bioMérieux, Marcy-l’Étoile, France), measuring 32.4 ng/L (reference <11 ng/L). Transthoracic echocardiography, however, demonstrated normal left ventricular size and function, with no regional wall-motion abnormalities, leaving the clinical significance of the biomarker elevation uncertain.

During a subsequent Emergency Department access, due to recurrent chest pain, coronary CT angiography was performed, revealing a moderate (40–50%) mid–left anterior descending (LAD) stenosis without additional significant lesions. A stress echocardiography excluded the presence of inducible ischemia during maximal workload. Continuous ECG monitoring showed nonspecific repolarization abnormalities, without evidence of arrhythmias or transient ischemic changes of functional significance. However, because troponin concentrations remained modestly but persistently elevated, invasive coronary angiography was recommended. Pre-procedural testing showed elevated high-sensitivity cardiac troponin I (hs-cTnI) concentrations (51.2 ng/L; Atellica^®^ IM, Siemens Healthineers, Erlangen, Germany; reference range 2.5–38.64 ng/L). Concurrent measurement of B-type natriuretic peptide (BNP) on the same analytical platform yielded values within the reference range (18 pg/mL; Atellica^®^ IM, Siemens Healthineers reference range 2–100 pg/mL), indicating no biochemical evidence of acute heart failure. In accordance with current recommendations for the diagnostic evaluation of patients presenting with chest discomfort, natriuretic peptides were also assessed to support the clinical interpretation of cardiac function [16,17,18,19,20]. Coronary angiography subsequently confirmed a 40–50% mid–left anterior descending (LAD) artery stenosis without significant disease in other vessels. Fractional flow reserve measured across the lesion was 0.99, definitively excluding hemodynamic significance. Following coronary angiography, N-terminal pro–B-type natriuretic peptide (NT-proBNP) was also assessed and remained within the reference range (114 pg/mL; Atellica^®^ IM, Siemens Healthineers reference range 0–125 pg/mL). A marked post-procedural rise in hs-cTnI (Atellica^®^ IM: 1240.7 ng/L) was observed, consistent with expected myocardial micro-injury following coronary instrumentation. Cardiovascular Magnetic Resonance (CMR) demonstrated normal biventricular morphology, preserved systolic function, and no evidence of inflammation or focal post-contrast delayed enhancement. At follow-up, the patient remained clinically stable, experiencing only mild exertional dyspnoea and no anginal symptoms. In the absence of structural or functional cardiac abnormalities, the cause of her chronically elevated troponin levels remained unexplained.

Due to persistently elevated high-sensitivity cardiac troponin I (hs-cTnI) concentrations over time a targeted stepwise analytical investigation for potential immunoassay interference was performed. As a first step, hs-cTn measurements were performed using the Atellica^®^ IM hs-TnI assay. Serum aliquots analyzed on this platform subsequently underwent antibody-capture pretreatment with specific heterophilic antibodies blocking tube (HBT, Scantibodies Laboratory Inc., Santee, CA, United States of America) to assess the potential impact of endogenous interfering antibodies on the immunoassay signal. A variation in measured hs-cTnI concentration greater than 20% after pretreatment was considered suggestive of antibody-mediated interference, in accordance with published recommendations [4,5].

Subsequently, PEG 6000 precipitation was performed as a routinely applicable method to screen for high–molecular-weight immunoglobulin-containing complexes. This approach selectively precipitates immunoglobulin-bound analyte while free troponin remains in the supernatant [14,15,21]. Post-PEG recovery values below 40% were interpreted as strongly suggestive of a precipitable troponin–immunoglobulin complex, in accordance with recent evidence on macrotroponin and antitroponin antibody interference [9,13,14].

Successively, a comprehensive laboratory investigation was undertaken to further characterize the persistent hs-cTnI elevation. Serum and lithium–heparin plasma samples were obtained by standard phlebotomy procedures, centrifuged, and processed within 2 h of collection. Aliquots intended for subsequent interference investigations and cross-platform comparison were stored at −20 °C and thawed only once immediately before analysis to minimize potential freeze–thaw effects. Selected aliquots were also analyzed at external reference laboratories using alternative immunoassay platforms; sample transportation was performed under controlled temperature conditions in accordance with established pre-analytical quality standards and manufacturers’ recommendations to ensure appropriate temperature control and traceability. Time to analysis and sample handling procedures were harmonized to ensure comparability of results across different immunoassay platforms. All procedures were performed according to the manufacturers’ instructions and internal laboratory standard operating procedures. Analyses at the originating laboratory were performed in an ISO 9001:2015 [22] (International Organization for Standardization (ISO): Geneva, Switzerland, Edition 5, 2015) –certified clinical laboratory operating under standardized quality assurance and internal quality control procedures. Samples were collected as part of routine clinical care, with written informed consent obtained for blood collection and laboratory testing, including consent for the use of leftover samples for research and publication purposes.

To provide orthogonal confirmation and to further characterize assay-dependent behavior, all available aliquots were analyzed using multiple commercial immunoassay platforms employing different antibody pairs and analytical architectures, including VIDAS^®^ High Sensitive Troponin I (bioMérieux, Marcy-l’Étoile, France), Atellica^®^ IM hs-TnI (Siemens Healthineers, Erlangen, Germany), Alinity^®^ hs-TnI (Abbott Diagnostics, Lake Forest, IL, United States of America), Stratus^®^ CS Acute Care Troponin I (Siemens Healthineers, Erlangen, Germany), and Cobas^®^ hs-TnT (Roche Diagnostics, Basel, Switzerland) [8,10,23].

**Figure 1:**
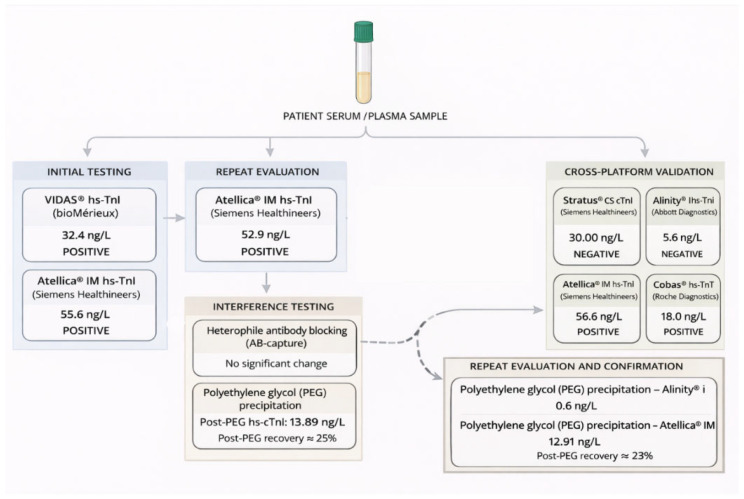
Schematic overview of the chronological laboratory evaluation performed, including cross-platform testing and interference analysis by heterophile antibody blocking and polyethylene glycol (PEG) precipitation.

A schematic overview of the analytical approach, illustrating the analysis of the same patient sample across different immunoassay platforms and interference-testing procedures, is shown in Figure 1. Analytical characteristics of the cardiac troponin immunoassays used, including limits of detection (LoD) derived from manufacturer documentation and assay characteristics, are summarized in Table 1. To further characterize potential sources of platform-dependent variability, the main technical and immunochemical differences among the cardiac troponin immunoassays used, including assay architecture, detection technology, antibody configuration, and epitope specificity—are summarized in Table 2.

A newly collected serum sample showed an hs-cTnI concentration of 55.6 ng/L when measured on the Atellica^®^ IM platform. Following treatment with heterophile antibody–blocking tube (HBT), hs-cTnI concentrations remained comparable to baseline values (52.94–54.59 ng/L), indicating no relevant effect of heterophile antibody interference.

In contrast, polyethylene glycol (PEG) precipitation resulted in a marked reduction of measurable hs-cTnI on the Atellica^®^ platform. Post-PEG concentrations decreased to 13.89 ng/L, corresponding to an approximate recovery of 25% strongly suggestive of a macrotroponin complex.

Repeat testing yielded consistent findings: hs-cTnI concentrations remained elevated on the Atellica^®^ platform (56.6 ng/L), whereas cardiac troponin I measured using the Stratus^®^ CS assay was negative according to assay cut-off (30 ng/L). PEG precipitation similarly resulted in a substantial reduction of the Atellica^®^ signal, with a post-PEG concentration of 12.91 ng/L (approximately 23% recovery).

Measurements obtained with the Alinity^®^ hs-cTnI assay were within the reference range (5.6 ng/L) and further decreased after PEG treatment (0.6 ng/L). Cardiac troponin T measured using the Cobas^®^ hs-cTnT assay was positive.

A summary of cardiac troponin measurements obtained across analytical platforms, including results before and after PEG precipitation, is reported in Table 3.

To further investigate the persistent elevation of cardiac troponin I observed during clinical follow-up, a structured laboratory evaluation was undertaken. The chronological sequence of the laboratory investigations is summarized in Figure 1.

Initial cardiac troponin I testing using the VIDAS^®^ hs-TnI assay revealed an increased concentration (32.4 ng/L). This finding was subsequently confirmed by measurement on the Atellica^®^ IM hs-cTnI platform, which also demonstrated elevated values (51.2 ng/L).

Further evaluation on a newly collected serum sample again showed elevated hs-cTnI concentrations on the Atellica^®^ IM platform (55.6 ng/L), confirming persistence of the abnormal finding over time. To assess the potential contribution of antibody-mediated assay interference, the sample underwent pretreatment with antibody-capture reagents. Following this procedure, hs-cTnI concentrations remained comparable to baseline values (52.94–54.59 ng/L), indicating no relevant change after heterophile antibody blocking.

**Figure 2:**
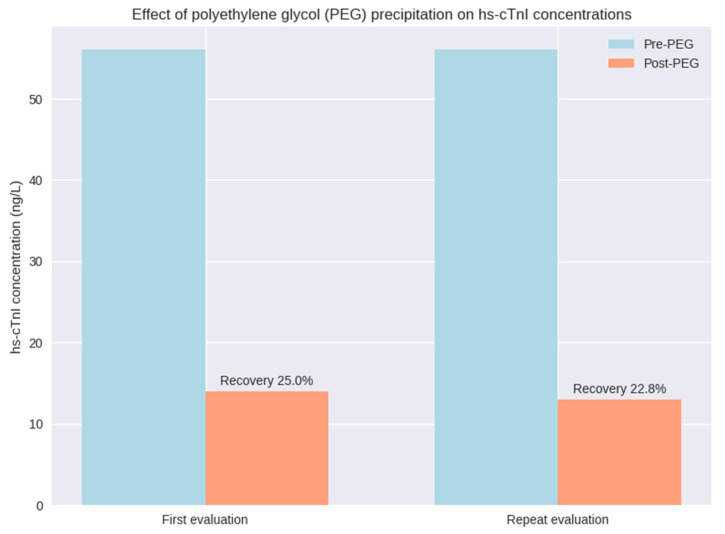
Effect of polyethylene glycol (PEG) precipitation on high-sensitivity cardiac troponin I (hs-cTnI) concentrations measured on the Atellica^®^ IM platform during the initial and repeat evaluations. Bars represent individual hs-cTnI measurements obtained before (Pre-PEG) and after PEG treatment (Post-PEG). Percentage values indicate post-PEG recovery relative to the corresponding pre-PEG concentration.

The same sample was subsequently subjected to polyethylene glycol (PEG) precipitation. This procedure resulted in a marked reduction of measurable hs-cTnI on the Atellica^®^ IM platform, with a post-PEG concentration of 13.89 ng/L, corresponding to an approximate recovery of 25%. The effect of polyethylene glycol (PEG) precipitation on hs-cTnI concentrations across analytical platforms is illustrated in Figure 2.

Repeated sampling and cross-platform analysis yielded consistent findings: hs-cTnI concentrations remained elevated on the Atellica^®^ IM platform (56.6 ng/L), whereas cardiac troponin I measured using the Stratus^®^ CS assay was below the limit of detection (30 ng/L). Measurements obtained with the Alinity^®^ i hs-TnI assay were within the reference range (5.6 ng/L) and further decreased following PEG precipitation (0.6 ng/L). PEG treatment again resulted in a substantial reduction of the Atellica^®^ hs-cTnI signal, with a post-PEG concentration of 12.91 ng/L (approximately 23% recovery). Cardiac troponin T measured using the Cobas^®^ hs-cTnT assay was positive.

## 3. Discussion

Persistent elevations of cardiac troponin in the absence of clinical, electrocardiographic, or imaging evidence of myocardial injury represent a well-recognized diagnostic challenge in contemporary clinical practice. While high-sensitivity cardiac troponin (hs-cTn) testing has substantially improved the early detection of myocardial injury, it remains susceptible to analytical interferences that may mimic chronic myocardial damage and lead to inappropriate diagnostic or therapeutic interventions.

In the present case, repeated hs-cTnI measurements showed a modest but persistent elevation confined to specific analytical platforms, with no rise-and-fall kinetic pattern and no concordant evidence of structural heart disease, inducible ischemia, or myocardial inflammation on comprehensive cardiologic evaluation. This biochemical–clinical discordance prompted a structured and systematic laboratory investigation to assess potential immunoassay interference.

Orthogonal testing across multiple immunoassay platforms revealed marked inter-assay discordance, with persistently elevated hs-cTnI concentrations on the index high-sensitivity assay, normal or undetectable values on alternative cTnI assays, and positive hs-cTnT [1,2,3,8]. Such platform-dependent variability strongly suggests epitope-specific detection of a circulating troponin complex rather than true free troponin released from injured cardiomyocytes, as previously described in cases of macrotroponin interference [9,10,11,24].

The observed platform-dependent variability can be explained by technical differences among cardiac troponin immunoassays, including assay architecture, detection technology, and antibody epitope specificity, which may influence the recognition of circulating troponin–immunoglobulin complexes such as macrotroponin. A comparative overview of these analytical characteristics is provided in Table 2.

Heterophile antibody interference was considered and systematically excluded using antibody-capture pretreatment, which did not produce any relevant change in measured hs-cTnI concentrations. In contrast, polyethylene glycol (PEG) precipitation resulted in a marked and reproducible reduction of detectable hs-cTnI, with post-PEG recoveries consistently below 30%. PEG-induced signal depletion is a recognized hallmark of high–molecular-weight immunoglobulin-containing complexes and supports the presence of macrotroponin I, an immunoglobulin-bound form of cardiac troponin that retains immunoreactivity while lacking biological activity [8,14,15].

Macrotroponin is an under-recognized, yet increasingly reported, cause of persistent and non-dynamic troponin elevation. Its detectability is highly assay-dependent and influenced by antibody specificity, epitope accessibility, and assay architecture, explaining the discordant results observed across platforms targeting different troponin epitopes or isoforms [8,9,21]. Reported prevalence of cardiac troponin autoantibodies varies widely and may be higher in patients with chronic inflammatory or autoimmune conditions, which may be relevant in the present case [11,13].

The analytical pattern observed—persistent elevation restricted to selected hs-cTnI assays, absence of heterophile antibody effects, marked PEG-related signal depletion, and normalization across orthogonal platforms—is characteristic of macrotroponin-related analytical interference. Importantly, these biochemical findings were fully concordant with the patient’s benign clinical course and the absence of structural or functional cardiac disease, thereby reconciling laboratory results with the overall clinical context.

Failure to recognize macrotroponin may lead to repeated testing, prolonged diagnostic uncertainty, and unnecessary or invasive cardiac investigations [7]. This case underscores the critical role of close collaboration between clinicians and laboratory specialists in the interpretation of cardiac troponin results in the presence of analytical interference. When troponin elevations are modest, persistent, assay-restricted, and inconsistent with the clinical presentation, systematic evaluation for analytical interference—including PEG precipitation and cross-platform comparison—should be incorporated into diagnostic pathway. This case highlights how appropriate laboratory investigation can resolve clinically incongruent troponin results and prevent misclassification of myocardial injury in the setting of analytical interference.

## 4. Conclusions

This case underscores the essential role of the clinical laboratory in resolving unexplained or discordant high-sensitivity cardiac troponin results in the presence of analytical interference. In the absence of clinical, functional, or imaging evidence of myocardial ischemia or structural heart disease, the patient’s persistent and modest hs-cTnI elevation initially generated significant diagnostic uncertainty, prompting multiple non-invasive and invasive cardiological evaluations. Despite an extensive work-up, all investigations were unremarkable and failed to identify any structural or functional cardiac abnormalities.

The identification of macrotroponin I, an immunoglobulin-bound troponin complex responsible for assay-dependent analytical interference, represented a decisive diagnostic turning point. Identification was supported by a consistent analytical pattern, including persistent elevation restricted to selected hs-cTnI platforms, lack of response to heterophile antibody-blocking procedures, marked signal reduction following polyethylene glycol precipitation, and normalization across orthogonal immunoassays. Correct recognition of this phenomenon reconciled the laboratory findings with the patient’s benign clinical course and prevented further unnecessary invasive interventions.

Importantly, recognition of analytical interference had a direct and meaningful impact on clinical management. Once macrotroponin was identified as the cause of the biochemical anomaly, clinicians were able to safely defer further invasive testing and repeated troponin measurements, thereby avoiding unnecessary procedures and patient anxiety. Patient management subsequently shifted from diagnostic escalation to a conservative, symptom-guided follow-up strategy, focusing on routine clinical assessment and optimization of cardiovascular risk factors rather than further invasive diagnostics.

During follow-up, the patient remained clinically stable, with no recurrence of anginal symptoms, no evidence of myocardial injury, and no adverse cardiovascular events, confirming that the persistent troponin elevation had no pathological significance. Given the widespread use of high-sensitivity troponin assays, systematic evaluation for analytical interferences—including macro-analytes such as macrotroponin—should be considered whenever troponin results are modestly elevated, non-dynamic, assay-restricted, and discordant with the clinical context. Awareness of this under-recognized source of interference is crucial to prevent misdiagnosis, reduce unnecessary interventions, optimize resource utilization, and support patient-centered, conservative management through appropriate integration of laboratory and clinical information.

## Figures and Tables

**Table 1:** Analytical characteristics of cardiac troponin immunoassays across analytical platforms.

Assay Platform	Analyte	Limit of Detection (LoD)	Manufacturer	Assay Characteristics
Atellica IM hs-TnI	cTnI	1.60 ng/L	Siemens Healthineers	High-sensitivity cardiac troponin I immunoassay
Alinity hs-TnI	cTnI	1.60 ng/L	Abbott Diagnostics	High-sensitivity cardiac troponin I immunoassay
VIDAS hs-TnI	cTnI	3.20 ng/L	bioMérieux	High-sensitivity cardiac troponin I assay based on enzyme-linked fluorescent immunoassay (ELFA) technology
Stratus CS	cTnI	30 ng/L	Siemens Healthineers	Non–high-sensitivity, previous-generation cardiac troponin I assay
Cobas hs-TnT	cTnT	3.16 ng/L	Roche Diagnostics	High-sensitivity cardiac troponin T assay

**Table 2:** Technical and immunochemical characteristics of cardiac troponin immunoassays.

Assay Platform	Assay Architecture	Detection Technology	Antibody Type	Epitope Specificity
Atellica^®^ IM hs-TnI	Three-site sandwich immunoassay	Chemiluminescence (acridinium ester label)	Monoclonal antibodies (mouse and sheep); Fab fragment used for detection	Capture antibody: aa 41–50, 171–190; Detection antibody: aa 29–34
Alinity^®^ hs-TnI	Two-step sandwich CMIA	Chemiluminescent microparticle immunoassay (acridinium label)	Monoclonal antibodies (mouse)	Not publicly disclosed
VIDAS^®^ hs-TnI	One-step sandwich ELFA	Enzyme-linked fluorescent immunoassay	Monoclonal antibodies (sandwich format; species not fully disclosed)	Capture antibody: aa 41–49, 24–40; Detection antibody: aa 87–95
Stratus^®^ CS cTnI	Two-site sandwich immunoassay (POC format)	Front-surface fluorescence (reflectance-based detection)	Monoclonal antibodies	Capture antibody: aa 27–32; Detection antibody: aa 41–56
Cobas^®^ hs-TnT	Two-site sandwich ECLIA	Electrochemiluminescence (ruthenium complex)	Monoclonal antibodies (mouse)	Capture antibody: aa 125–131; Detection antibody: aa 136–147

Information on assay architecture and epitope specificity was obtained from IFCC reference materials (2021–2024) and manufacturers’ analytical characterization documents, where publicly available. Abbreviations: CMIA, chemiluminescent microparticle immunoassay; ELFA, enzyme-linked fluorescent assay; ECLIA, electrochemiluminescence immunoassay; POC, point-of-care.

**Table 3:** Cardiac troponin measurements across platforms before and after interference testing.

Platform	Manufacturer	Sample/Treatment	Result	Units	Reference Range
VIDAS^®^ hs-TnI	bioMérieux	Serum	32.4	ng/L	<11
Atellica^®^ IM hs-TnI	Siemens Healthineers	Serum	51.2	ng/L	2.5–38.64
Cobas^®^ hs-TnT	Roche Diagnostics	Serum	18	ng/L	<14
Atellica^®^ IM hs-TnI	Siemens Healthineers	Serum	55.6	ng/L	2.5–38.64
Atellica^®^ IM hs-TnI	Siemens Healthineers	AB-capture	52.94/ 54.59	ng/L	2.5–38.64
Atellica^®^ IM hs-TnI	Siemens Healthineers	PEG-treated	13.89	ng/L	2.5–38.64
Stratus^®^ CS cTnI	Siemens Healthineers	Li-heparin plasma	30 ng/L	ng/L	< 60.00
Atellica^®^ IM hs-TnI	Siemens Healthineers	Serum	56.6	ng/L	2.5–38.64
Atellica^®^ IM hs-TnI	Siemens Healthineers	PEG-treated	12.91	ng/L	2.5–38.64
Alinity^®^ i hs-TnI	Abbott Diagnostics	Serum	5.6	ng/L	<15.6
Alinity^®^ i hs-TnI	Abbott Diagnostics	PEG-treated	0.6	ng/L	<15.6

High-sensitivity cardiac troponin I (hs-cTnI) and cardiac troponin T (hs-cTnT) concentrations measured using different immunoassay platforms are shown for native samples and after heterophile antibody–blocking treatment (AB-capture) or polyethylene glycol (PEG) precipitation. Results are reported in the units specified by each manufacturer.

## Data Availability

The original contributions presented in this study are included in the article. Further inquiries can be directed to the corresponding author(s).
